# Bioactivity of Two Polyphenols Quercetin and Fisetin against Human Gastric Adenocarcinoma AGS Cells as Affected by Two Coexisting Proteins

**DOI:** 10.3390/molecules27092877

**Published:** 2022-04-30

**Authors:** Bo Wang, Jing Wang, Xin-Huai Zhao

**Affiliations:** 1 College of Pharmacy, Heilongjiang University of Chinese Medicine, Harbin 150040, China; wangbohljzyy@163.com; 2 Centre Testing International Pinbiao (Jiangsu) Certification Technology Co., Ltd., Nanjing 210046, China; neauwj@163.com; 3 Key Laboratory of Dairy Science, Ministry of Education, Northeast Agricultural University, Harbin 150030, China; 4 School of Biological and Food Engineering, Guangdong University of Petrochemical Technology, Maoming 525000, China; 5 Research Centre of Food Nutrition and Human Healthcare, Guangdong University of Petrochemical Technology, Maoming 525000, China

**Keywords:** quercetin, fisetin, protein, gastric cancer cells, anticancer bioactivity

## Abstract

It is recognized that minor dietary components polyphenols have anticancer effects on digestive tract, lung, leukemia, and other cancers, while polyphenols also can covalently or noncovalently interact with major dietary components proteins such as casein, soybean proteins, whey proteins, and bovine serum albumin. Thus, whether the noncovalent interaction between the molecules of two polyphenols (quercetin and fisetin) and two proteins (bovine serum albumin and casein) has positive or negative impact on anticancer activities of the polyphenols against human gastric adenocarcinoma AGS cells was assessed in this study. The two polyphenols had obvious anticancer activities to the cells, because dose levels as low as 20–160 μmol/L caused reduced cell viability of 30.0–69.4% (quercetin) and 24.6–63.1% (fisetin) (using a cell treatment time of 24 h), or 9.9–48.6% (quercetin) and 6.4–29.9% (fisetin) (using a cell treatment time of 48 h). However, the cell treatments by the polyphenols in the presence of the two proteins mostly caused lower polyphenol activity toward the cells, compared with those treatments by the polyphenols in the absence of the proteins. Specifically, the presence of the proteins led to reduced growth inhibition in the cells, because higher cell viability of 33.2–86.7% (quercetin) and 29.1–77.7% (fisetin) at 24 h, or 14.1–66.8% (quercetin) and 7.9–59.0% (fisetin) at 48 h, were measured in these treated cells. The two coexisting proteins also yielded the polyphenol-treated cells with less mitochondrial membrane potential loss, less formation of reactive oxygen species, and decreased cell apoptosis. Thus, it is highlighted that the noncovalent interaction between dietary polyphenols and proteins resulted in weakened anticancer ability for the polyphenols to the gastric cancer cells.

## 1. Introduction

Polyphenols are natural dietary components and ubiquitous in plant foods such as strawberry, apple, onion, grape, tea, and coffee [1,2,3]. Numerous studies have shown that polyphenols have multiple bioactivities, including the antioxidant, antimicrobial, antiinflammatory, and anticancer, together with immune function and cardiovascular protection [4,5,6,7,8]. Currently, plant-derived chemopreventive and chemotherapeutic agents receive increasing worldwide attention due to their nontoxic characteristics and fewer side effects in the body [9]. Two dietary polyphenols, namely quercetin and fisetin, have been regarded to have anticancer ability towards liver, breast, prostate, and colon carcinoma in vivo and in vitro. Su and coauthors found that when bladder cancer cells (T24, UMUC3, and MB49) were exposed to quercetin, the expression levels of p-AMPK and p-p70s6k were upregulated and downregulated, respectively, revealing that quercetin induced cell apoptosis via activating the AMPK signaling pathway [10]. Meanwhile, it was reported that quercetin showed antiproliferative activity in a dose- and time-dependent manner against breast cancer BT-474 cells, and could induce cell apoptosis through the caspase-dependent extrinsic pathway [11]. It was also observed that fisetin had an ability to suppress proliferation, migration, adhesion, and invasion of the cancer A549 cells, or induce cell apoptosis via targeting the extracellular signaling pathway with activation on caspase-3 and caspase-9 [12]. In addition, it was also revealed that fisetin may induce cell apoptosis in human renal carcinoma cells by inducing caspase activation, PARP cleavage, and DR5 upregulation [13]. In the study of Ekström and coauthors, higher intake of dietary quercetin was identified to be negatively associated with the risk of noncardia gastric cancer [14]. Two other studies also reported that fisetin could reduce the proliferation of human gastric cancer SGC7901 cells and induce cell apoptosis [15], or was able to suppress the growth of human gastric carcinoma AGS and SNU-1 cells and induced cell apoptosis via ROS-mediated mitochondrial pathway [16]. In our diets, many food components such as proteins, polysaccharides, lipids, polyphenols, among others, are taken into the stomach simultaneously. Although both quercetin and fisetin are regarded to have anticancer activities with several gastric cancer cells, whether coexisting substances such as proteins have potential impact on the anticancer activities of quercetin and fisetin still remains less investigated. Thus, such a study deserves our consideration.

It is well known that the proteins as the main food components usually can interact with the minor food components such as polyphenols noncovalently, which yields the noncovalent interaction between proteins and polyphenols [17]. As expected, this noncovalent interaction causes property changes for both proteins and polyphenols. Al-Hanish and coauthors evaluated the noncovalent interactions between bovine α-lactalbumin (ALA) and epigalocatechin-3-gallate (EGCG), the polyphenol from green tea [18]. The results from far-UV circular dichroism spectroscopy showed that this noncovalent interaction altered secondary structure of ALA with increased β-sheet but decreased α-helix content [18]. It was also reported that the noncovalent interaction between soy protein isolate (SPI) and curcumin caused higher antioxidant activity in the SPI-curcumin complex that was formed, compared with SPI only [19]. In addition, it was revealed that quercetin-loaded bovine serum albumin nanoparticles had better free radical scavenging activity than quercetin itself [20]. However, when milk was mixed with various teas such as Darjeeling, green, and English breakfast teas, a decrease in the antioxidant capacities of the teas was observed [21]. Chemically, catechins in the teas could complex noncovalently with milk proteins such as β-casein, which leads to a lower number of free hydroxyl groups. As a consequence, the teas were detected with lower antioxidant activity. More importantly, when the adenocarcinoma cells (HT-29) were treated with EGCG and the casein-EGCG complex, the complex at higher doses (0.97 and 1.95 μmol/L) showed lower antiproliferative ability than EGCG of the same doses, obviously indicating that the noncovalent association between casein micelles and EGCG finally induced declined cytotoxic effect of EGCG on the HT-29 cells [22]. Meanwhile, a contrary conclusion was found in other research [23], in which the noncovalent interaction between plumbagin and human serum albumin (HSA)-endowed plumbagin with enhanced cytotoxicity and cell apoptosis in HeLa cells. Proteins as one of the major components in foods can inevitably and noncovalently interact with polyphenols, and thus affect the anticancer activity of polyphenols. A study assessing whether two proteins, namely bovine serum albumin (BSA) and casein, could affect the anticancer activities of quercetin and fisetin against the AGS cells is thereby necessary.

This study exposed AGS cells to the two polyphenols, quercetin and fisetin, in the presence or absence of two proteins BSA and casein, and then for the first time identified the effects of the two targeted proteins on anticancer activities of the two polyphenols via assaying the following indices: growth inhibition, cell apoptosis, mitochondrial membrane potential (MMP) loss, and formation of intracellular reactive oxygen species (ROS). The aim of this study was to reveal whether and how the noncovalent polyphenol–protein interaction affected the anticancer function of the two polyphenols in the stomach.

## 2. Materials and Methods

### 2.1. Chemicals and Kits

Both fisetin and quercetin with purity greater than 98% were purchased from Shanghai Yousi Biotechnology Co. Ltd. (Shanghai, China) and Dalian Meilun Biotechnology Co. Ltd. (Dalian, China), respectively. Dimethyl sulfoxide (DMSO) and BSA were obtained from Solarbio Science and Technology Co. Ltd. (Beijing, China). Casein (protein content of 88.93% on dry basis) was purchased from Beijing Aoboxing Bio-Tech Co. Ltd. (Beijing, China). Ultrapure water was used while other chemicals were of analytical grade.

Cell Counting Kit-8 (CCK-8) was obtained from Dojindo Laboratories (Dojindo Laboratories, Kyushu, Japan). The ROS assay kit, Annexin V-FITC apoptosis detection kit, and JC-1 were purchased from Beyotime Biotechnology (Shanghai, China).

### 2.2. Cell Culture

Human gastric adenocarcinoma cell line (i.e., AGS cells) was purchased from Cell Bank of the Chinese Academy of Sciences (Shanghai, China). The cells were required to be cultured in RPMI-1640 complete medium (HyClone, Logan, UT, USA) supplemented with 10% fetal bovine serum (FBS) (HyClone), 100 U/mL penicillin and streptomycin, and kept at 37 °C in a humidified atmosphere containing 5% CO_2_. Subconfluent cells (about 80%) were then passaged with 0.25% trypsin solution containing 0.02% EDTA.

### 2.3. Assay of Cell Viability

AGS cells were seeded in 96-well plates at a density of 1 × 10^4^ cells per well and incubated at 37 °C. After 24 h, different doses of fisetin and quercetin (20, 40, 80, 120, 160 μmol/L), alone or in combination with casein or BSA at a concentration of 1.0 g/L in the medium, were added into the cells and incubated for 24–48 h. Control cells were also treated with 0.1% DMSO, 1.0 g/L BSA, or casein in 0.1% DMSO medium, respectively. The cells treated by 100 μmol/L 5-Fu only served as a positive. After incubation for 24 and 48 h at 37 °C, the cells were washed three times with PBS (0.01 mol/L, pH 7.0), while CCK-8 (final concentration of 10%) of 10 μL was added into each well. The cells were incubated for another 2 h, while the absorbance was measured by a microplate reader (Bio Rad Laboratories, Hercules, CA, USA) at a wavelength of 450 nm. The control cells were served with cell viability of 100%, as previously described [24]. In addition, IC_50_ value was calculated based on the relationship between the assessed polyphenol dose and resultant growth inhibition, as previous described [25].

### 2.4. Morphology Assessment

The cells were seeded in 6-well plates (1 × 10^4^ cells per 100 μL per well) for attachment. Afterward, the media were discarded, and 0.1% DMSO, 1.0 g/L BSA/casein, and 80 μmol/L quercetin/fisetin with or without 1.0 g/L BSA/casein was applied on the cells at 37 °C for 24 h. The cells were rinsed with the PBS twice, while 4% paraformaldehyde in PBS was added to fix the cells at 4 °C overnight. After cell washing with PBS twice, Hoechst 33,258 of 0.5 mL was added to stain the cells for 5 min. The cells were rinsed with PBS twice, while the images were observed and captured by a fluorescence microscope (Olympus, Tokyo, Japan) with excitation and emission wavelengths of 350 and 460 nm, respectively.

### 2.5. Measurement of Mitochondrial Membrane Potential

To measure the changes of mitochondrial membrane potential (MMP, ∆*Ψ*_m_), the cells were seeded in 6-well plates at a density of 1 × 10^4^ cells per 100 μL per well for attachment. Then, the cells were inoculated with 0.1% DMSO, 1.0 g/L BSA/casein, or 80 μmol/L quercetin/fisetin with or without 1.0 g/L BSA/casein at 37 °C for 24 h. After that, the cells were stained with the fluorescent probe JC-1 dye (10 μmol/L) in the dark at 37 °C for 20 min, washed twice with ice-cold PBS, and analyzed by a flow cytometry (FACS Calibur, Becton Dickson, San Jose, CA, USA). The fluorescence intensities were measured at 527 nm (green) and 590 nm (red), as previously described [26]. Cell-Quest software vision 5.1 (D Biosciences, Franklin Lakes, NJ, USA) was used for data analysis.

### 2.6. Cell Apoptosis Assay

Apoptotic cells were detected using the Annexin V-FITC apoptosis detection kit, as previously described [27]. In brief, the cells were seeded at a density of 5 × 10^5^ per 60 mm plate overnight to allow to adhere. The cells were treated with 0.1% DMSO, 1.0 g/L BSA/casein, or quercetin/fisetin (80 μmol/L) with and without the proteins (1.0 g/L) at 37 °C for 24 h. All cells were collected, washed by the PBS, stained with FITC-conjugated annexin-V and PI in binding buffer in the dark at 20 °C for 15 min, and then analyzed using flow cytometry. The cells in early apoptotic (Q_4_) and late apoptotic (Q_2_) phases were used to calculate total apoptotic cells, which is expressed as percentage [27].

### 2.7. Measurement of Intracellular ROS

The intracellular ROS was measured using the fluorescent probe dichlorofluorescein–diacetate (DCFH–DA), as previously described [28]; the cells were seeded at a density of 5 × 10^5^ per 60 mm plate overnight to adhere, treated with 0.1% DMSO and 1.0 g/L BSA/casein, quercetin, and fisetin (80 μmol/L) with and without the proteins (1.0 g/L) at 37 °C for 24 h. The cells were washed with the PBS and incubated with 10 μmol/L DCFH-DA at 37 °C for 30 min in the dark. The DCF fluorescence of the cells was measured by a Hitachi F-4500 fluorescence spectrometer (Tokyo, Japan). A slit width 0.5 nm and wavelengths at 488 nm (excitation) or 525 nm (emission) were used in this measurement.

### 2.8. Statistical Analysis

All experiments and analyses were performed in triplicate. The data were analyzed using SPSS 16.0 software (SPSS, Inc., Chicago, IL, USA), and expressed as means ± standard deviations. One-way ANOVA analyses of variance followed by Duncan’s multiple range test was used to determine the differences between the means, and were analyzed (*p* < 0.05).

## 3. Results

### 3.1. Growth Inhibition of the Polyphenols on the Cells

**Figure 1:**
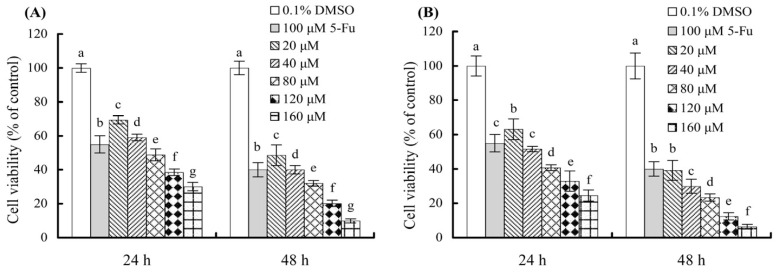
Measured viability values of the cells treated with 0.1% DMSO, 100 μmol/L 5-Fu, 20–160 μmol/L quercetin (**A**) and fisetin (**B**) for 24 and 48 h. Different lowercase letters above the columns of the same treatment time indicate that one-way ANOVA of the mean values is significantly different (*p* < 0.05).

When the cells were treated for 24 or 48 h with various doses of quercetin and fisetin (20–160 μmol/L), the cells showed decreased viability values dose-dependently (Figure 1), indicating that both quercetin and fisetin could inhibit the growth of AGS cells. With a cell treatment time of 24 h, quercetin and fisetin at 20–160 μmol/L induced viability values of 30.1–69.4% and 24.6–63.1%, with the calculated IC_50_ values about 64.2 and 43.4 μmol/L, respectively (Table 1). With a longer treatment time of 48 h, quercetin and fisetin at the same doses resulted in viability values of 9.9–48.6% and 6.4–39.2%, with decreased IC_50_ values of 21.4 and 12.8 μmol/L, respectively (Table 1). These data demonstrated three facts—the two polyphenols assessed had inhibitory activities on the AGS cells, fisetin had higher activity than quercetin in the cells, and most importantly, longer treatment time of the cells with the polyphenols consistently induced stronger inhibition on the cells.

### 3.2. Growth Inhibition of the Polyphenols on the Cells as Affected by the Coexisting Proteins

**Figure 2:**
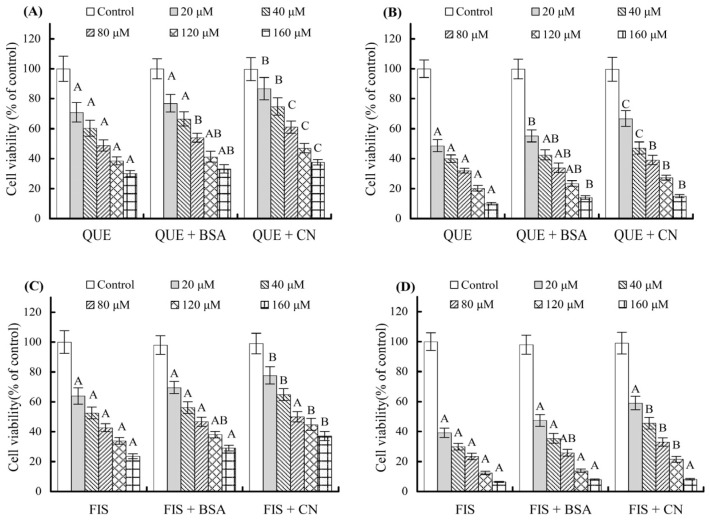
The effects of bovine serum albumin (BSA) and casein (CN) on the growth inhibition of quercetin (QUE) and fisetin (FIS) on the cells with treatment times of 24 h (**A**,**C**) and 48 h (**B**,**D**). Different uppercase letters in the columns of the same polyphenol dose indicate that one-way ANOVA of the mean values is significantly different (*p* < 0.05).

When the polyphenols coexisting with the two proteins at 1 g/L were used to treat the cells, the cells were detected to have higher viability values than those treated with the polyphenols alone (Figure 2). When using a cell treatment time of 24 h, quercetin at 20–160 μmol/L brought about viability values of 33.2–77.0% (with BSA) and 37.6–86.7% (with casein), while fisetin at the same doses caused viability values of 29.1–69.6% (with BSA) and 37.1–77.7% (with casein). In this case, quercetin and fisetin consistently caused higher cell viability values, thereby estimated to have enhanced IC_50_ values of 69.3–79.6 and 48.7–52.1 μmol/L, respectively (Table 1). Similarly, when using a cell treatment time of 48 h, quercetin caused viability values of 14.1–55.2% (with BSA) and 15.1–66.8% (with casein) while fisetin led to viability values of 7.9–47.4% (with BSA) and 8.2–59.0% (with casein). Thus, quercetin and fisetin were calculated to have IC_50_ values of 25.8–31.4 and 17.1–21.4 μmol/L, respectively (Table 1). It is thus seen clearly from the calculated data given in Table 1 that both quercetin and fisetin in the presence of the two proteins had decreased growth inhibition on the cells, while casein was more active than BSA to attenuate the growth inhibition of the two polyphenols. This finding revealed the negative impact of the two coexisting proteins (or the noncovalent protein–polyphenol interaction) on the assessed polyphenol bioactivity. Furthermore, it could be seen that a polyphenol dose of 80 μmol/L generally led to growth inhibition near or more than 50% (Table 1). This dose was then used in later assays in this study.

**Figure 3:**
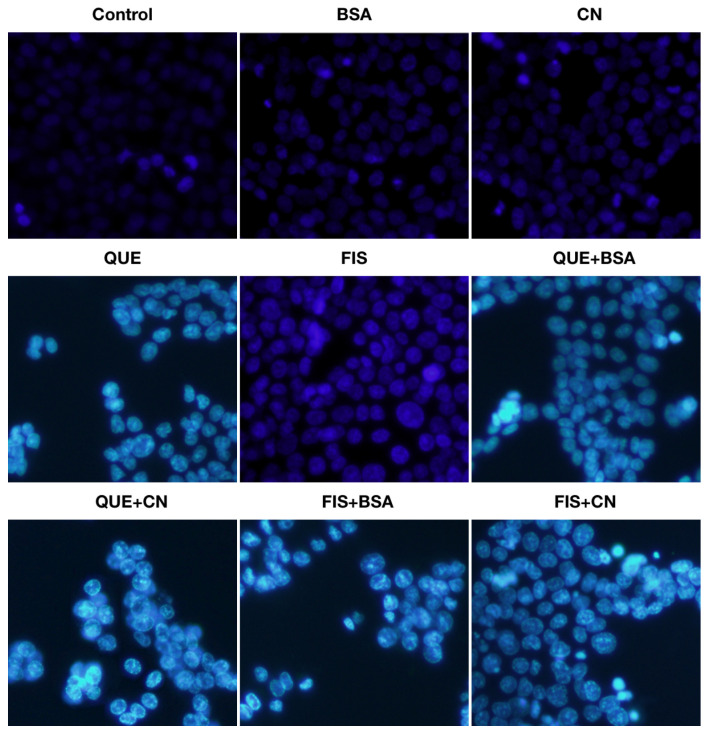
Morphological features of the cells treated with 0.1% DMSO (control), quercetin (QUE), fisetin (FIS), bovine serum albumin (BSA), casein (CN), and the polyphenol–protein mixtures for 24 h. The polyphenol dose was 80 μmol/L, while the protein dose was 1.0 g/L.

Furthermore, a brief observation of cell morphological changes in these cells exposed to different treatments may provide evidence for the effect of the two coexisting proteins on polyphenol activity. Hoechst 33,258 staining results (Figure 3) showed that 0.1% DMSO, 1.0 g/L BSA or casein did not affect cell morphology. However, the polyphenol-treated cells were spindle-shaped with diffuse homogeneous fluorescence. To be more specific, the cells treated with quercetin or fisetin in the absence of the two proteins had fragmented nuclei and apoptotic bodies with dense particle lump fluorescence. This fact indicated that the two polyphenols caused cell apoptosis. Meanwhile, those cells treated with quercetin or fisetin in the presence of the two proteins showed less cell apoptosis, suggesting the two proteins possessed an ability to attenuate the cell apoptosis of the assessed polyphenols.

### 3.3. MMP Loss of the Cells as Affected by the Two Polyphenols and Coexisting Proteins

**Figure 4:**
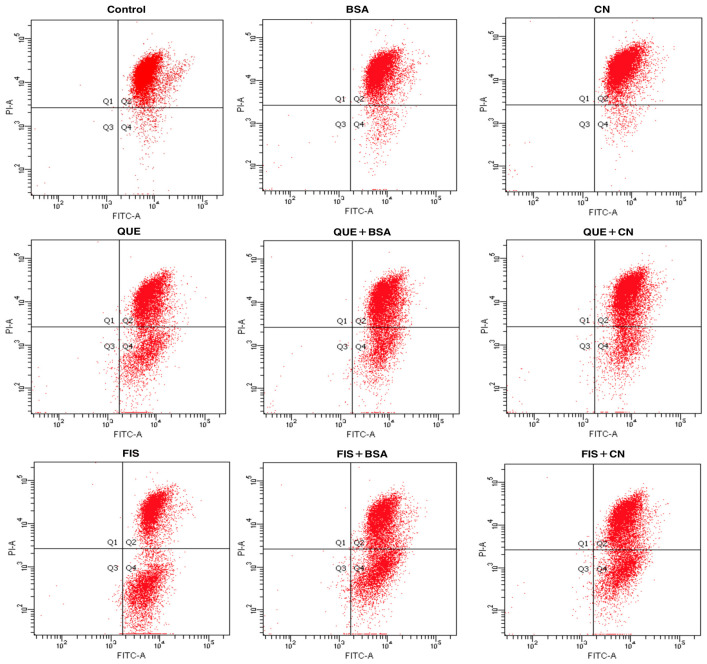
Flow cytometric results of the mitochondrial potential of the cells treated with 0.1% DMSO (control), quercetin (QUE), fisetin (FIS), bovine serum albumin (BSA), casein (CN), and polyphenol–protein mixtures. Q_2_ illustrates J-aggregates in functional (polarized) mitochondria that emit red fluorescence, while Q_4_ illustrates monomers in resting (depolarized) mitochondria that emit green fluorescence. The polyphenol dose was 80 μmol/L, while the protein dose was 1.0 g/L.

The JC-1 probe was used to measure MMP of the treated cells by a flow cytometry technique (Figure 4). In brief, the cells in quadrant 2 (Q_2_) (i.e., fluorescence red) had higher MMP while those cells in Q_4_ (i.e., fluorescence green) possessed MMP loss. The results indicated that about 95.7% of the control cells were in Q_2_, while 96.2% and 95.4% of the cells exposed to casein and BSA, respectively, were in Q_2_. This fact meant that the two proteins did not have any significant impact on MMP loss, leading to more cells in Q_4_ (or lower ratio of fluorescence red to fluorescence green). However, when the cells were exposed to fisetin and quercetin at 80 μmol/L for 24 h, they were measured with Q_2_ values of 54.0% and 70.1%, respectively, demonstrating that the two compounds, and especially fisetin, led to clear MMP loss in the cells. Unfortunately, in the presence of casein and BSA, a cell treatment with 80 μmol/L fisetin for 24 h resulted in respective Q_2_ values of 65.0% and 56.4%, while the other cell treatment with 80 μmol/L quercetin for 24 h caused Q_2_ values of 79.9% and 74.0%, respectively. It was observed that the two proteins, especially casein, had an ability to inhibit the bioactivity of the two coexisting polyphenols toward the cells, causing a weaker potential to decline cell MMP. All these results again confirmed that the two coexisting proteins (or the noncovalent protein–polyphenol interaction) also had a negative impact on anticancer effect of fisetin and quercetin in the cells, while casein was more active than BSA in inhibiting polyphenol activity.

### 3.4. The Two Proteins Attenuated Apoptosis Induction of the Two Polyphenols in AGS Cells

**Figure 5:**
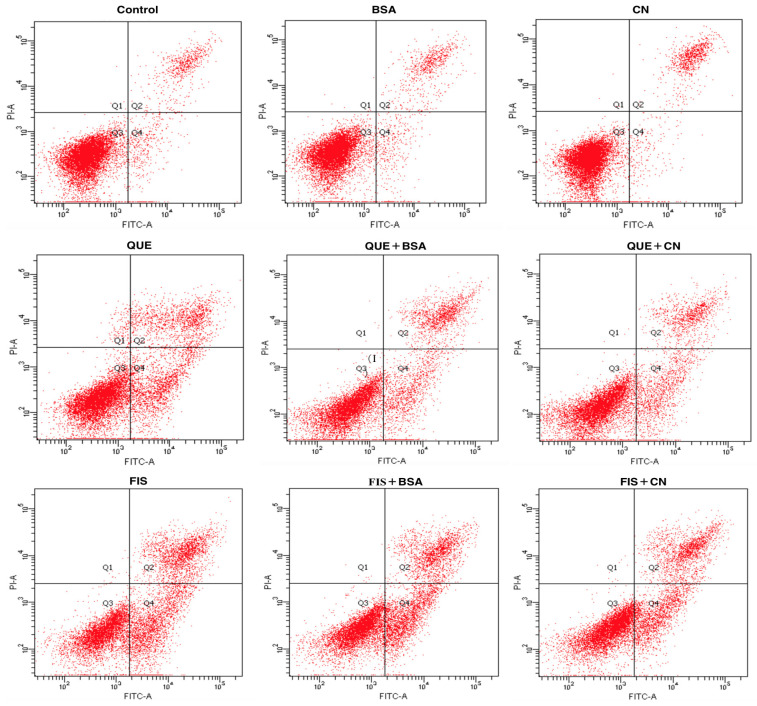
Identification of the normal and apoptotic cells using the Annexin V/propidium iodide (PI) staining and flow cytometry. The cells were treated with 0.1% DMSO (control), quercetin (QUE), fisetin (FIS), bovine serum albumin (BSA), casein (CN), and the polyphenol–protein mixtures. The polyphenol dose was 80 μmol/L, while the protein dose was 1.0 g/L.

Annexin V/propidium iodide (PI) staining was used to detect apoptotic cells in the AGS cells after different treatments for 24 h (Figure 5). The image was divided into four quadrants (Q_1_–Q_4_), representing the healthy (Q_3_), early apoptotic (Q_4_), late apoptotic (Q_2_), and necroptotic (Q_1_) cell populations, respectively. In general, the normal cells cannot be stained either by Annexin V or PI; because of cell membrane integrity, the fluorescent dye cannot enter the cells. When cells are stimulated by various substances, the phosphatidyl serine translocates from the inner to the outer leaflet of the cell membrane, and then binds to Annexin V. More importantly, at late apoptotic or early necroptotic stage, cell membrane is damaged and PI penetrates to bind to the DNA. The cells undergoing necrosis rather than apoptosis can only be stained by PI. The data listed in Table 2 show the average percentages of calculated cell populations in these cells with different treatments. When the cells were exposed to 0.1% DMSO, BSA, and casein, the respective percentages of total apoptotic cells (Q_2_ + Q_4_) were 10.2%, 11.1%, and 11.3%. Meanwhile, the cells with quercetin and fisetin treatments showed increased percentages of total apoptotic cells of 32.3% and 50.0%, respectively. These data confirmed that that quercetin and fisetin had apoptosis induction in AGS cells, while fisetin was more potent than quercetin in the cells. However, in the presence of BSA and casein, the treated cells had decreased total apoptotic percentages of 25.5% (quercetin with BSA), 28.7% (quercetin with casein), 45.0% (fisetin with BSA), and 38.0% (fisetin with casein). That is, both BSA and casein suppressed the apoptosis induction of quercetin and fisetin to the cells, leading to reduced percentages of total apoptotic cells. In other words, the noncovalent interaction between the two polyphenols and two coexisting proteins was proved to inhibit the anticancer potentials of quercetin and fisetin to the cells.

### 3.5. Prooxidation of the Two Polyphenols in the Cells as Affected by the Two Proteins

**Figure 6:**
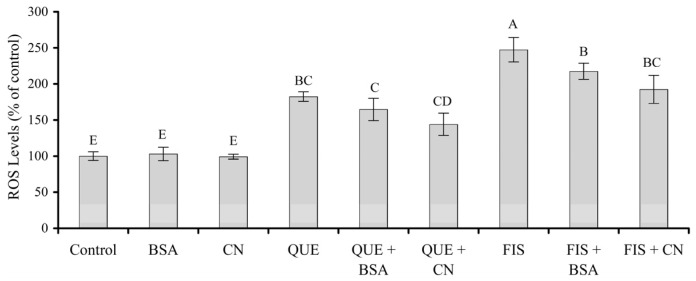
Measured intracellular ROS levels in the control cells treated by 0.1% DMSO, or those treated by quercetin (QUE) and fisetin (FIS) in the absence or presence of bovine serum albumin (BSA) or casein (CN). The polyphenol dose was 80 μmol/L, while the protein dose was 1.0 g/L. Different uppercase letters above the columns indicate that one-way ANOVA of the mean values is significantly different (*p* < 0.05).

Cells have a perfect system to produce and scavenge the intracellular ROS to maintain vital redox balance. Once the balance is broken, the overload ROS are harmful to cell structure and function, leading to these adverse events such as DNA damage, lipid peroxidation, and protein oxidation. In cancer cells, ROS level is relatively high. This makes the cancer cells more vulnerable than the normal cells to oxidative stress-induced cell death. Intracellular ROS levels of these cells with various treatments were also examined in this study. The results obtained (Figure 6) demonstrated that the intracellular ROS levels were significantly increased to 182% and 247% in response to quercetin and fisetin treatments, respectively, while BSA and casein had no impact on ROS formation in the cells because the measured ROS levels were about 99% and 102%. However, the cell exposure with quercetin or fisetin in the presence of BSA, especially casein, resulted in a decrease in ROS levels. To be more specific, quercetin with BSA or casein caused ROS levels of 144–165%, while fisetin with BSA or casein yielded ROS levels of 192–217%. Thus, casein was more potent than BSA in reducing the prooxidation of quercetin or fisetin in the cells. Collectively, it was thus evidenced that the noncovalent protein–polyphenol interaction inhibited prooxidation of the two polyphenols in the cells.

## 4. Discussion

Food matrixes naturally are rich in bioactive components and nutraceuticals; together with major components such as polysaccharides and proteins [29], these compounds may interact with each other through various pathways. The scientific community agrees that proteins can bind with polyphenols via various interaction forces such as hydrogen bonds, hydrophobic interaction, electrostatic force, and van der Waals forces [30]. It is known now that the noncovalent interaction between polyphenols and proteins may generally induce various property changes for both polyphenols and proteins. For example, when human blood plasma or albumin were mixed with quercetin, rutin, and catechin, the measured total antioxidant capacity of the three polyphenols was lower than that of the free polyphenols themselves, indicating the proteins masked the antioxidation of the polyphenols [31]. BSA also could mask the antioxidant activities of flavonoids such as quercetin, morin, fisetin, myricetin, kaempferol, galangin, and catechin, because the targeted flavonoid–BSA complexes were not easily oxidized compared to flavonoids alone [32]. It was also clarified that the stability of fisetin and quercetin in solutions was enhanced by the coexisting proteins, due to the existing noncovalent protein–polyphenol interaction [33], while the noncovalent interaction between tea polyphenols and soy protein yielded an improvement in emulsion ability and emulsion stability [34]. When wheat and chickpea flour or soy protein isolate were mixed with blueberry polyphenols to form polyphenol–protein aggregates, this noncovalent interaction led to higher antioxidation in the lipopolysaccharide-injured RAW 264.7 cells by reducing NO and ROS generation [35]. Moreover, in the presence of polyphenols, whey proteins at pH 6 formed smaller particles with higher net charge much more easily, while the gels had lower gel point and hardness [36]. From a chemical point of view, the protein–polyphenol interaction yields noncovalent binding of polyphenols into the proteins, thereby less polyphenols are free or available. Subsequently, the noncovalent protein–polyphenol interaction reasonably induces lower bioactivity for the assessed polyphenols. In the present study, the two polyphenols had a noncovalent interaction with the added BSA or casein, and thus provided fewer free molecules toward the cells. The polyphenols in the presence of the two coexisting proteins thereby exerted lower anticancer effects on the cells, including weaker proliferation inhibition, failure to cause morphology change and MMP loss, reduced proportion of apoptotic cells, and lower ROS generation in the cells. The present results also proposed that special attention should be given to these coexisting major food components, especially proteins, when aiming to assess the bioactivities of these phytochemicals or nutraceuticals contained in food matrixes, because of the unavoidable noncovalent interaction between proteins and phytochemicals.

Meanwhile, differing chemical features of polyphenols and proteins have an impact on the resultant noncovalent protein–polyphenol interaction, such as the critical binding affinity. Xiao and coauthors investigated the binding affinity of 50 dietary polyphenols to bovine milk proteins, and demonstrated that the binding affinity of the polyphenols to bovine milk proteins was related to the differences of polyphenol structure [37]. When kaempferol and quercetin noncovalently interacted with casein, it was found that quercetin with two −OH groups at the B-ring had higher affinity to caseinate than kaempferol only with one −OH group at the B-ring [38]. In addition, a previous study also reported that the energy changes (ΔG) for the covalent interaction between galangin and whey proteins at 20–40 °C ranged from −32.8 to −35.9 kJ/mol, with calculated apparent binding parameters of (6.96–9.64) × 10^5^ L/mol [39]. Another study also found that the energy changes (ΔG) for the noncovalent interaction between galangin and casein at the same temperature scales ranged from −33.3 to −36.4 kJ/mol, while the calculated apparent binding parameters were up to (8.83–11.80) × 10^5^ L/mol [40]. Clearly, in the two studies [39,40], casein showed a greater ability than whey proteins to bind with galangin, resulting in higher binding parameter and more energy decrease. Chemically, casein is regarded to exhibit higher hydrophobicity than whey proteins; for example, the major casein fractions have hydrophobicity values of 4.7–5.6 kJ/residue while BSA has a hydrophobicity value of only 4.3 kJ/residue [41]. Thus, casein can interact with the polyphenols strongly, because hydrophobic interaction usually is the main driving force involved in the noncovalent protein–polyphenol interaction [40]. In the present study, it was reasonable that casein interacted much more efficiently with the two polyphenols than BSA; thus, the two polyphenols in the presence of casein showed much reduction in the assessed anticancer effects on the cells.

Currently, anticancer effects of natural substances are assessed via measuring indices such as cell morphology, viability, MMP loss, ROS levels, apoptosis, cell invasion, DNA fragments and gene expression at transcription and translation levels, among others [42]. For example, quercetin was revealed to have anticancer effects on human papillary thyroid cancer B-CPAP cells via growth inhibition, cell apoptosis, and caspase activation [43], or exert anticancer ability in the nonsmall cell lung cancer A549 cells via the induction of mitotic catastrophe [44]. Quercetin could also inhibit cell migration by disassembling microfilaments and inducing apoptosis in A549 cells [44]. Meanwhile, it was also found that fisetin at 20–80 μmol/L could inhibit the proliferation of breast cancer cells (4T1, MCF-7, and MDA-MB-231) and the invasion or migration of 4T1 cells, and possess apoptosis induction to 4T1 cells [45]. Hsieh and coauthors also documented the anticarcinoma activity of fisetin against the human renal cell cells such as 786-O, A-498, Caki-1, and ACHN cells [46]. Therefore, the present study employed growth inhibition, apoptosis induction, ROS formation, and MMP loss as the critical indices to reveal how the targeted noncovalent protein–polyphenol interaction caused a negative impact on these anticancer activities of the two polyphenols against the AGS cells. Why fisetin had a higher anticancer potential in the cells than quercetin may relate to their different molecular features. It has been proposed that the fewer −OH groups contained in a compound, the smaller polarity of the compound [47]. Subsequently, the compound with lower polarity can interact easily with the cell membrane to exert its bioactivity [48]. Fisetin shares the same chemical structure with quercetin in both B- and C-rings, but they have different −OH group numbers in the A-ring (one versus two −OH groups). Fisetin, having four −OH groups in its molecules, thus possesses lower polarity than quercetin, which has five −OH groups in its molecules. Therefore, in this study, fisetin exerted greater bioactivity in AGS cells than quercetin. Similar results were reported in two previous studies, in which quercetin was found to have higher barrier-promoting ability in the IEC-6 cells than myricetin (with six −OH groups in its molecules) [49], or galangin (with three −OH groups in its molecules) was more active than kaempferol (with four −OH groups in its molecules) in improving the barrier function of IEC-6 cells [48]. It is thus acceptable that polyphenol bioactivity is governed by their chemical structures, especially the −OH group numbers in their molecules.

## 5. Conclusions

This study highlighted an unavoidable but possibly neglected effect of coexisting proteins on the in vitro anticancer properties of two polyphenols, fisetin and quercetin, in human gastric adenocarcinoma AGS cells. Although the two polyphenols, especially fisetin, showed clear anticancer activities to the cells by causing proliferation inhibition, ROS formation, MMP loss, and apoptosis induction, the presence of BSA and casein consistently brought about decreased polyphenol activity in the cells. To be more specific, the two coexisting proteins resulted in the polyphenols having lower capacity to perform proliferation inhibition, enhance ROS generation, injure cell membrane, and induce cell apoptosis. It was thus proved that the noncovalent interaction between the proteins and polyphenols had a negative impact on the anticancer properties of the assessed polyphenols in the cells. It was also noticeable that, compared with BSA, casein was a more potent agent to reduce polyphenol bioactivity in the cells. The present results thus addressed that other food components in diets should be considered for their possible impact on the bioactivities of the assessed dietary biomolecules when a noncovalent interaction between the biomolecules and food components is unavoidable.

## Figures and Tables

**Table 1:** Calculated IC_50_ values (μmol/L) of the two polyphenols in the AGS cells with treatment times of 24 and 48 h in the presence or absence of bovine serum albumin (BSA) and casein (CN).

Treatment Time	Quercetin		Fisetin	
	Without Proteins	With Proteins	Without Proteins	With Proteins
24 h	64.2 ± 4.8	69.3 ± 3.2 (BSA) 79.6 ± 2.6 (CN)	43.4 ± 3.7	48.7 ± 4.4 (BSA) 52.1 ± 2.8 (CN)
48 h	21.4 ± 1.9	25.8 ± 1.4 (BSA) 31.4 ± 3.6 (CN)	12.8 ± 1.1	17.1 ± 4.9 (BSA) 22.4 ± 2.5 (CN)

**Table 2:** Proportions of the viable (Q_3_) and apoptotic (Q_2_ + Q_4_) cells detected in AGS cells subjected to different treatments for 24 h in the presence or absence of bovine serum albumin (BSA) and casein (CN).

Cell Group	Q_3_ (%)	Q_2_ + Q_4_ (%)
0.1% DMSO	88.7	10.2
BSA	88.9	11.1
Casein	88.7	11.3
Quercetin	66.5	32.3
Quercetin + BSA	74.5	25.5
Quercetin + CN	71.2	28.7
Fisetin	49.9	50.0
Fisetin + BSA	54.9	45.0
Fisetin + CN	61.9	38.0

## Data Availability

All data are contained within the article.

## Sample Availability

Samples of the compounds including quercetin and fisetin are available from the authors.
